# Late device-related thrombus after left atrial appendage closure following early antithrombotic discontinuation: The paradox of complete sealing

**DOI:** 10.1016/j.hroo.2026.04.012

**Published:** 2026-04-20

**Authors:** Osamu Hashimoto, Hayata Uesako, Yukiyoshi Tashima, Yasuo Tokoro

**Affiliations:** 1 Department of Cardiology, Shin-Kuki General Hospital, Kuki, Saitama, Japan; 2 Tokoro Cardiology and Internal Medicine Clinic, Koshigaya, Saitama, Japan

**Keywords:** Left atrial appendage closure, Device-related thrombus, Peridevice leak, Computed tomography, Implantation depth, Spontaneous echo contrast, Left atrial appendage emptying velocity, Antithrombotic discontinuation

## Abstract

**Background:**

Device-related thrombus (DRT) after left atrial appendage closure can occur.

**Objective:**

This study aimed to evaluate late/very-late DRT after an institutional early “all-off” strategy guided by complete sealing (peridevice leak [PDL] 0).

**Methods:**

We analyzed 106 consecutive successful left atrial appendage closure procedures; 103 patients had evaluable imaging at 45 days (≥45 days). Per protocol, antithrombotic agents were discontinued at 45 days and/or 6 months if imaging showed PDL of 0 and no DRT. Landmark analyses in patients free of 45-day DRT (n = 101) compared late/very-late DRT (6–12 months) between all-off and continued-therapy groups.

**Results:**

DRT occurred in 6 of 103 patients (5.8%): 2 at 45 days and 4 at 6–12 months. Late/very-late DRT occurred in 4 of 33 (12.1%) in the all-off group vs 0 of 68 (0.0%) in the continued-therapy group (*P* = .010). A sensitivity analysis restricted to patients with PDL of 0 and no 45-day DRT (n = 44) showed a consistent direction (4 of 28 vs 0 of 16; *P* = .280). Among patients with PDL of 0 and no 45-day DRT who continued direct oral anticoagulants (n = 8), the primary reason was recent ischemic stroke at <6 months (6 of 8; 75%).

**Conclusion:**

Reliance on PDL of 0 alone to guide early complete withdrawal may be insufficient in selected patients; findings are hypothesis generating.

## Graphical abstract

**Figure:**
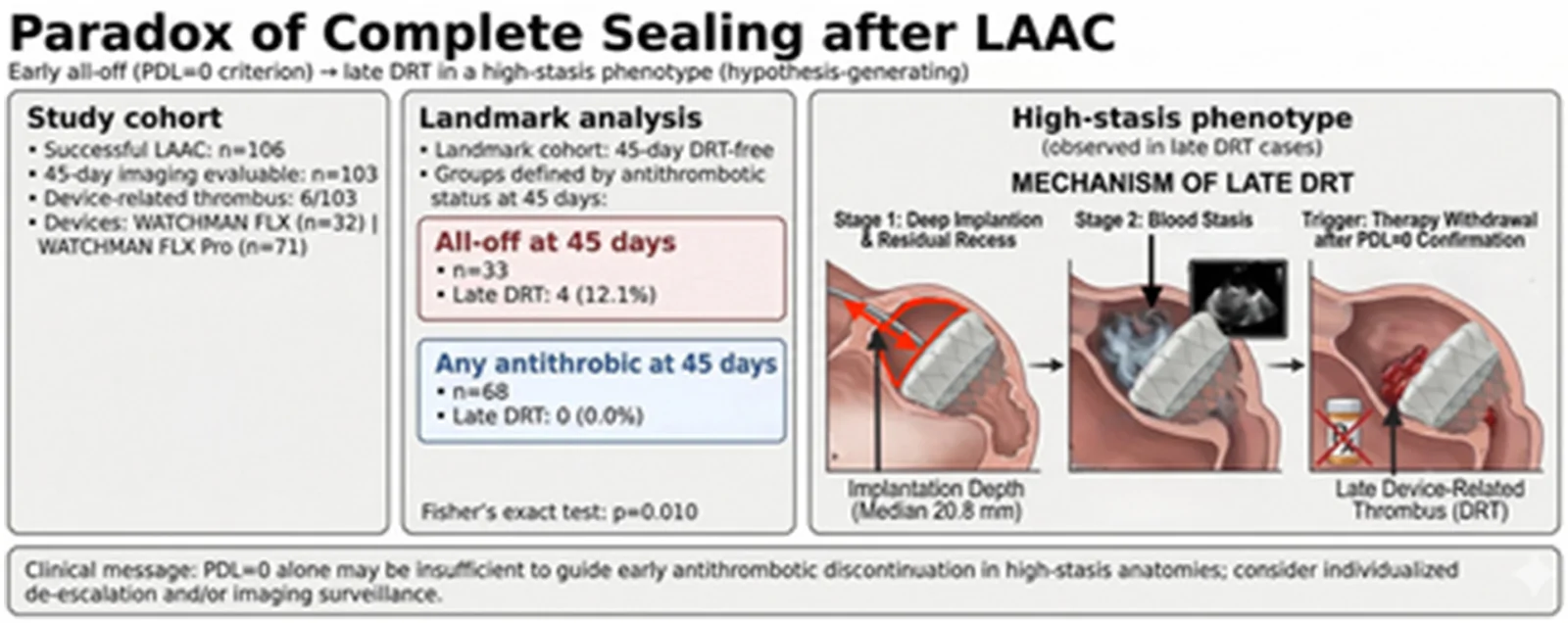

**Key Findings**

▪ Device-related thrombus (DRT) was detected in 5.8% of patients with evaluable 45-day imaging after left atrial appendage closure, including 2 early and 4 late/very-late events.
▪ Late/very-late DRT occurred exclusively in patients who discontinued all antithrombotic therapy at 45 days, whereas no events were observed in patients who remained on therapy at 45 days.
▪ DRT clustered in a high-stasis phenotype characterized by deeper implantation and baseline appendage stasis features (dense spontaneous echo contrast and low left atrial appendage emptying velocity).
▪ Complete sealing (peridevice leak 0) alone was insufficient to ensure safety for early full antithrombotic withdrawal in selected high-stasis anatomies, supporting physiology-guided de-escalation strategies.

## Introduction

Percutaneous left atrial appendage (LAA) closure (LAAC) is an established alternative to long-term oral anticoagulation for stroke prevention in selected patients with atrial fibrillation (AF). Device-related thrombus (DRT), although infrequent, is clinically important because it has been associated with thromboembolic events and often mandates intensification of antithrombotic therapy.^1^ Implantation technique and postprocedural antithrombotic strategy are key, modifiable contributors to DRT risk.^2^

A growing body of evidence suggests that deeper device implantation may create a residual “neo-appendage” recess proximal to the device, potentially predisposing to blood stasis and DRT.^3^ Conversely, clinical practice commonly emphasizes the achievement of “complete sealing” (no peridevice leak [PDL]) as a prerequisite for de-escalating therapy.^2^ We hypothesized a potential paradox: in certain anatomic configurations—particularly when implantation depth is substantial—complete sealing may transform the residual recess into a closed, low-washout chamber such that late thrombosis becomes apparent after complete withdrawal of pharmacologic protection.

Using a single-center registry with computed tomography (CT)–dominant follow-up and a protocol favoring early discontinuation of all antithrombotic therapy (“all-off”) once complete sealing was confirmed, we aimed to (1) describe the incidence and timing of DRT, with a focus on late/very-late events after therapy withdrawal, and (2) characterize the relationship among implantation depth, baseline stasis markers (including LAA emptying velocity [LAAEV] and spontaneous echo contrast [SEC]), and late/very-late DRT.

## Methods

### Study design, setting, and ethics

This was a single-center observational cohort study based on a prospectively maintained institutional LAAC registry. We included consecutive patients undergoing successful percutaneous LAAC between June 1, 2023, and November 30, 2025. The administrative censoring date (data lock) was November 30, 2025. The study was approved by the Shin-Kuki General Hospital Ethics Committee (no. 126). A written informed consent was obtained from all patients as part of institutional participation in the nationwide J-LAAO registry (mandatory at our institution for LAAC procedures), which includes consent for the use of deidentified data for research. All data were deidentified before analysis, and all images used in figures were fully anonymized. The research reported in this paper adhered to the Declaration of Helsinki.

### Population and analytical cohorts

A total of 106 procedures achieved successful LAAC. All procedures were performed using the WATCHMAN FLX or WATCHMAN FLX Pro device (Boston Scientific, Marlborough, MA). For DRT incidence and time-to-detection descriptions, we analyzed patients with evaluable follow-up imaging at the first surveillance visit (45-day visit; target 45 days with an allowance window of ±30 days). Therefore, the denominator for DRT incidence was 103 patients with evaluable 45-day imaging. Patients without 45-day imaging were excluded from DRT incidence analyses (they were not assumed to be free of DRT).

### Follow-up schedule and imaging adjudication

Surveillance imaging was scheduled at (1) 45 days after LAAC (target 45 days ±30; corresponding to 15–75 days after the index procedure), (2) 6 months after LAAC (±90 days; 90–270 days), and (3) 12 months after LAAC (−90 to +180 days; 271–540 days). Cardiac CT was the preferred imaging modality; transesophageal echocardiography (TEE) was used when CT was not performed, particularly in patients with severe renal dysfunction (estimated glomerular filtration rate <30 mL/min/1.73 m^2^). CT findings were based on formal institutional radiology reports. TEE was performed and interpreted by a Japanese Board of Perioperative Transesophageal Echocardiography–certified anesthesiologist together with cardiologists with expertise in periprocedural TEE.

### Definitions

DRT was defined as a thrombus adherent to the atrial aspect of the device detected on follow-up CT or TEE. Early DRT refers to DRT detected at the 45-day visit; late/very-late DRT refers to DRT detected beyond the 45-day visit (typically at 6 or 12 months).^1^^,^^2^

PDL was measured in mm at each follow-up imaging. In the pivotal WATCHMAN trials, discontinuation of oral anticoagulation at 45 days was guided by the absence of device thrombus and minimal or no peridevice flow (jet ≤5 mm width).^4^ In our registry, complete sealing was defined as PDL of 0 mm at the relevant follow-up visit. Because antithrombotic de-escalation decisions were made at the 45-day visit (and again at 6 months), the primary landmark analyses adjudicated complete sealing using the 45-day PDL value; PDL measurements at subsequent visits are reported descriptively in the case table and Supplemental Materials.

**Figure 1:**
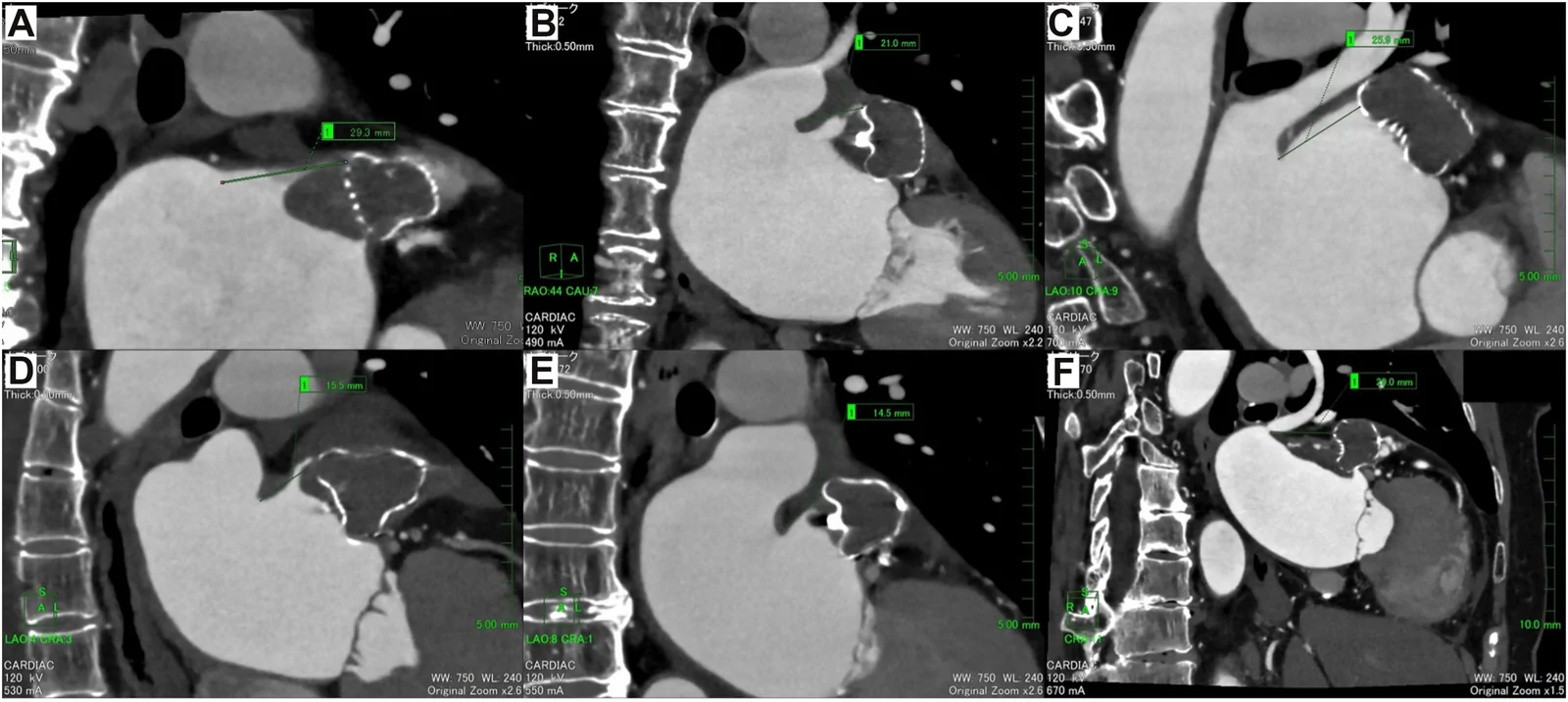
CT-based measurement of implantation depth. Representative oblique CT views demonstrating measurement of implantation depth from the pulmonary vein limbus to the atrial device surface. Substantial depth may create a residual proximal recess (neo-appendage/cul-de-sac). The oblique plane is intended to illustrate depth measurement; an eccentric device-related thrombus may not be fully visualized in this plane. CT = computed tomography.

Implantation depth was measured on 45-day CT as the distance from the pulmonary vein limbus to the atrial surface of the device (Figure 1). Deep implantation was defined as >10 mm, consistent with previous literature^3^; because this threshold was common in the present cohort, implantation depth was also evaluated as a continuous measure and using exploratory higher thresholds. Measurements were performed retrospectively by 1 investigator using standardized multiplanar reconstruction; inter- and intraobserver variability were not formally assessed.

Baseline appendage stasis markers were obtained from preprocedural TEE: LAAEV (cm/s) and SEC. SEC severity was graded semiquantitatively from 0 (none) to 4+ (severe) and was dichotomized as dense vs nondense for analysis.^5^ Low LAAEV was defined a priori as ≤20 cm/s.

AF pattern was classified as paroxysmal vs nonparoxysmal (persistent or permanent) in the source dataset.

### Antithrombotic protocol and safety override

Institutional post-LAAC management prioritized early de-escalation of antithrombotic therapy once device-related complications were excluded. In principle, if follow-up imaging at the 45-day and/or 6-month visit demonstrated both absence of DRT and complete sealing (PDL 0), all antithrombotic agents were discontinued (“all-off”), and patients were not routinely switched to single antiplatelet therapy. Exceptions (“safety override”) included patients with an established indication for antiplatelet therapy (eg, coronary, cerebrovascular, or peripheral artery disease) or perceived high thromboembolic risk (eg, recent ischemic stroke within 6 months), in whom continued anticoagulation or antiplatelet therapy was permitted at the treating physician’s discretion.^6^^,^^7^

From implantation to the first surveillance imaging visit, antithrombotic therapy (typically oral anticoagulation with or without antiplatelet therapy) was prescribed according to institutional practice and the treating physician’s judgment. For the landmark analyses, antithrombotic status was defined at the 45-day visit based on medications being taken at the time of follow-up imaging. “All-off at 45 days” indicates that no oral anticoagulant or antiplatelet agent was being taken at the 45-day imaging visit; “any antithrombotic at 45 days” indicates continued use of any oral anticoagulant and/or antiplatelet therapy. Patients who remained on therapy at 45 days could subsequently undergo further de-escalation after the 6-month visit if DRT was absent and PDL of 0, unless another indication persisted; however, group assignment for the 45-day landmark analyses was fixed at 45 days.

### Statistical analysis

Categorical variables are presented as counts (percentages) and compared using Fisher’s exact test. Continuous variables are presented as medians [interquartile ranges] and compared using the Mann–Whitney U test. Because DRT events were infrequent, we focused on descriptive analyses and prespecified landmark comparisons rather than multivariable modeling. A landmark analysis at the 45-day visit was performed among patients free of DRT at 45 days (n = 101), comparing late/very-late DRT incidence between those who discontinued all antithrombotic therapy at 45 days (“all-off”) and those who remained on any antithrombotic therapy. A sensitivity analysis further restricted the landmark cohort to patients with complete sealing at 45 days (PDL 0). Exploratory subgroup analyses examined whether baseline stasis markers modified the association between all-off withdrawal and late/very-late DRT. A 2-sided *P* < .05 was considered statistically significant.

## Results

### Study cohort

Among 106 successful LAAC procedures, 103 patients had evaluable 45-day follow-up imaging and comprised the analytical cohort for DRT incidence. All evaluable 45-day surveillance studies in this cohort were performed using cardiac CT. Baseline characteristics are presented in Table 1. Devices used were WATCHMAN FLX (n = 32) and WATCHMAN FLX Pro (n = 71). The cohort had high thromboembolic and bleeding risk profiles (median CHA_2_DS_2_-VASc score of 6.0 and HAS-BLED score of 3.0). Maintenance hemodialysis was present in 13 patients (12.6%).

**Table 1:** Baseline and procedural characteristics (updated dataset)

Characteristic	Overall (N = 103)	DRT (n = 6)	No DRT (n = 97)	*P* value
Age, y, median (IQR)	78.0 (73.0–82.0)	80.5 (79.2–82.5)	77.0 (73.0–82.0)	.087
CHA_2_DS_2_-VASc score, median (IQR)	6.0 (5.0–7.0)	7.0 (7.0–7.0)	6.0 (5.0–7.0)	.012
HAS-BLED score, median (IQR)	3.0 (3.0–4.0)	4.0 (3.2–4.0)	3.0 (3.0–4.0)	.167
LAAEV, cm/s, median (IQR)	23.0 (17.0–39.0)	18.5 (15.5–20.0)	24.0 (18.0–40.0)	.03
Device type (WATCHMAN FLX Pro), n (%)	71 (68.9)	4 (66.7)	67 (69.1)	1.000
Female sex, n (%)	30 (29.1)	4 (66.7)	26 (26.8)	.058
Nonparoxysmal AF (persistent/permanent), n (%)	62 (60.2)	6 (100.0)	56 (57.7)	.079
Hemodialysis, n (%)	13 (12.6)	0 (0.0)	13 (13.4)	1.0
Dense SEC, n (%)	64 (62.1)	6 (100.0)	58 (59.8)	.08
Implantation depth at 45-d CT, mm, median (IQR)	12.6 (9.2–17.6)	20.8 (19.8–24.6)	12.1 (9.1–17.2)	.001
Deep implantation (>10 mm), n (%)	72 (69.9)	6 (100.0)	66 (68.0)	.175
Extreme implantation depth (>20 mm), n (%)	16 (15.5)	4 (66.7)	12 (12.4)	.005
Complete sealing at the 45-d visit (PDL 0), n (%)	46 (44.7)	6 (100.0)	40 (41.2)	.007
High-stasis phenotype (depth >10 + LAAEV ≤20 + dense SEC), n (%)	31 (30.1)	6 (100.0)	25 (25.8)	.001
Previous stroke/TIA/systemic embolism, n (%)	76 (73.8)	6 (100.0)	70 (72.2)	.336

*P* values are unadjusted and provided for descriptive purposes only.

AF = atrial fibrillation; CT = computed tomography; DRT = device-related thrombus; IQR = interquartile range; LAAEV = left atrial appendage emptying velocity; PDL = peridevice leak; SEC = spontaneous echo contrast; TIA = transient ischemic attack.

### Incidence and timing of DRT

DRT was identified in 6 of 103 patients (5.8%). DRT occurred with both device generations (WATCHMAN FLX 2 of 32; WATCHMAN FLX Pro 4 of 71). 2 cases were detected at the 45-day visit, and 4 were detected at 6–12 months (late/very-late DRT). A representative CT depiction of DRT is shown in Supplemental Figure 1. Clinical and imaging characteristics of DRT cases are presented in Table 2.

**Table 2:** Clinical and imaging characteristics of DRT cases

Case	DRT detected	DRT size (maximum, mm)	Implantation depth (mm)	Baseline stasis (LAAEV, cm/s; SEC)	AF type	45-d status (ATT; PDL)	Status at DRT detection (ATT; PDL)	Concomitant events
Case 1	45-d (CT)	18.3	20.8	20 cm/s; dense	Permanent	Dabigatran 220 mg; PDL 0	Dabigatran 220 mg; PDL 0	None
Case 2	45-d (CT)	22.4	25.9	20 cm/s; dense	Persistent	Edoxaban 15 mg; PDL 0	Edoxaban 15 mg; PDL 0	None
Case 3	12-mo (CT)	7.4	20.8	14 cm/s; dense	Permanent	None; PDL 0	Edoxaban 30 mg; PDL 0.0	Ischemic stroke (timing relative to DRT detection not adjudicated)
Case 4	6-mo (CT)	41.3	19.5	15 cm/s; dense	Persistent	None; PDL 0	Apixaban 10 mg; PDL 0	None
Case 5	6-mo (CT)	57.4	29.3	20 cm/s; dense	Permanent	None; PDL 0	Apixaban 10 mg; PDL 0	None
Case 6	6-mo (CT)	13.9	16.8	17 cm/s; dense	Permanent	None; PDL 0	Edoxaban 30 mg; PDL 0	None

DRT size represents the maximum dimension measured on CT. Antithrombotic status and PDL are reported at the time of each follow-up imaging assessment and at the time of DRT detection.

AF = atrial fibrillation; ATT = antithrombotic therapy; CT = computed tomography; DRT = device-related thrombus; LAAEV = left atrial appendage emptying velocity; PDL = peridevice leak; SEC = spontaneous echo contrast.

### Baseline phenotype associated with DRT

Patients who developed DRT had higher CHA_2_DS_2_-VASc scores than those who did not (median 7.0 vs 6.0; *P* = .012) (Table 1). Implantation depth at 45-day CT was greater in patients with DRT than in those without (20.8 [19.8–24.6] vs 12.1 [9.1–17.2]; *P* = .001). Baseline LAAEV was lower in patients with DRT (18.5 [15.5–20.0] vs 24.0 [18.0–40.0]; *P* = .030). All DRT cases had nonparoxysmal AF (6 of 6; 100%) and dense SEC (6 of 6; 100%), whereas these features were present in 56 of 97 (59.8%) and 58 of 97 patients without DRT (59.8%), respectively (both *P* ≈ .08). Using binary definitions, deep implantation (>10 mm) was universal in DRT cases (6 of 6; 100%) but also common among patients without DRT (66 of 97; 68.0%; *P* = .175), indicating limited discrimination by the 10-mm cutoff in this cohort. By contrast, an exploratory threshold of >20 mm was enriched among DRT cases (4 of 6; 66.7%) compared with patients without DRT (12 of 97; 12.4%; *P* = .005).

### Landmark analysis: Antithrombotic discontinuation and late/very-late DRT

**Figure 2:**
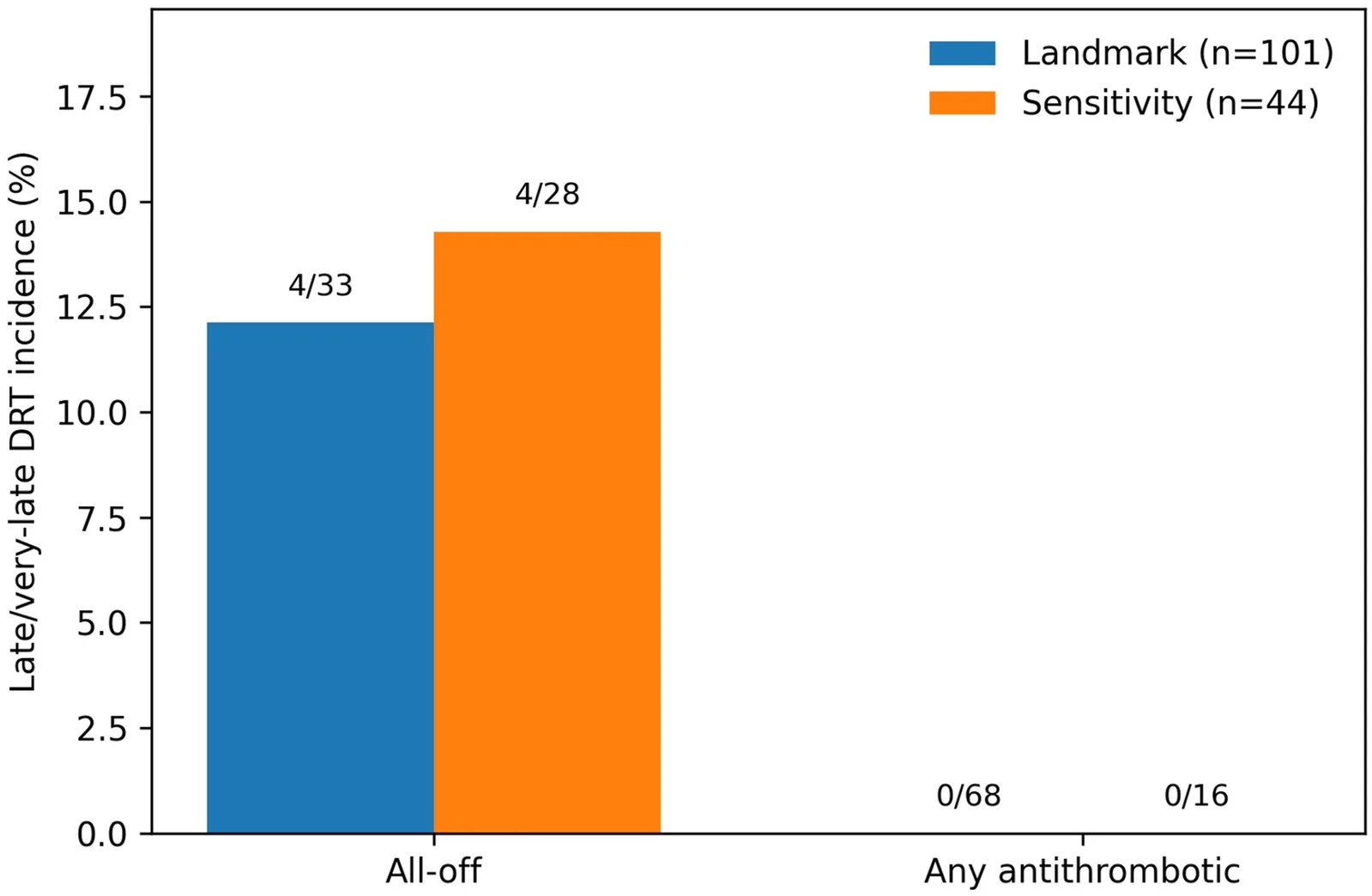
Late/very-late DRT incidence by 45-day antithrombotic status. Bar plot of late/very-late DRT (6–12 months) among patients free of DRT at the 45-day visit, stratified by all-off vs any antithrombotic therapy at 45 days. A sensitivity cohort restricted to complete sealing (peridevice leak 0) is shown for comparison. DRT = device-related thrombus.

In the landmark cohort restricted to patients free of DRT at 45 days (n = 101), 33 patients were off all antithrombotic therapy at the 45-day visit (“all-off”), including 28 with complete sealing (PDL 0) and 5 with residual PDL, whereas 68 patients remained on any antithrombotic therapy. Although the institutional protocol also allowed complete discontinuation after the 6-month visit if DRT was absent and PDL of 0, all late/very-late DRT events occurred in patients who were already all-off at the 45-day landmark (ie, none occurred in the continued-therapy group). Late/very-late DRT occurred in 4 of 33 (12.1%) in the all-off group and in 0 of 68 (0.0%) in the continued-therapy group (Fisher’s exact *P* = .010) (Table 3 and Figure 2).

**Table 3:** Landmark and sensitivity analyses for late/very-late DRT

Analysis	Group	N	Late/very-late DRT (6–12 mo)	Rate (%)	*P* value (Fisher’s exact)
Landmark (DRT-free at the 45-d visit)	All-off at 45 d	33	4	12.1	.010
Landmark (DRT-free at the 45-d visit)	Any antithrombotic at 45 d	68	0	0.0	
Sensitivity (PDL of 0 and DRT-free at the 45-d visit)	All-off at 45 d	28	4	14.3	.280
Sensitivity (PDL of 0 and DRT-free at the 45-d visit)	Any antithrombotic at 45 d	16	0	0.0	

DRT = device-related thrombus; PDL = peridevice leak.

The continued-therapy group at the 45-day landmark was enriched for residual PDL (52 of 68; 76.5%) compared with the all-off group (5 of 33; 15.2%). Among patients with complete sealing (PDL 0) and no 45-day DRT who nonetheless remained on therapy (n = 16), continuation of a direct oral anticoagulant despite complete sealing (“safety override”) occurred in 8 (50.0%), most often owing to recent ischemic stroke within 6 months (6 of 8; 75.0%). These patterns underscore the potential for confounding by indication in observational comparisons.

Follow-up imaging at 6 and/or 12 months was available in 86 of 101 landmark patients (85.1%), and all of these follow-up studies were also performed using cardiac CT. Follow-up imaging availability was similar between groups (29 of 33 [87.9%] [all-off] vs 57 of 68 [83.8%] [any antithrombotic]; *P* = .768).

In the sealing-restricted sensitivity analysis limited to patients with PDL of 0 and no 45-day DRT (n = 44), late/very-late DRT occurred in 4 of 28 (14.3%) among those who discontinued all antithrombotic therapy and in 0 of 16 (0.0%) among those maintained on any antithrombotic therapy (Fisher’s exact *P* = .280), showing a consistent direction but limited statistical power owing to few events.

**Figure 3:**
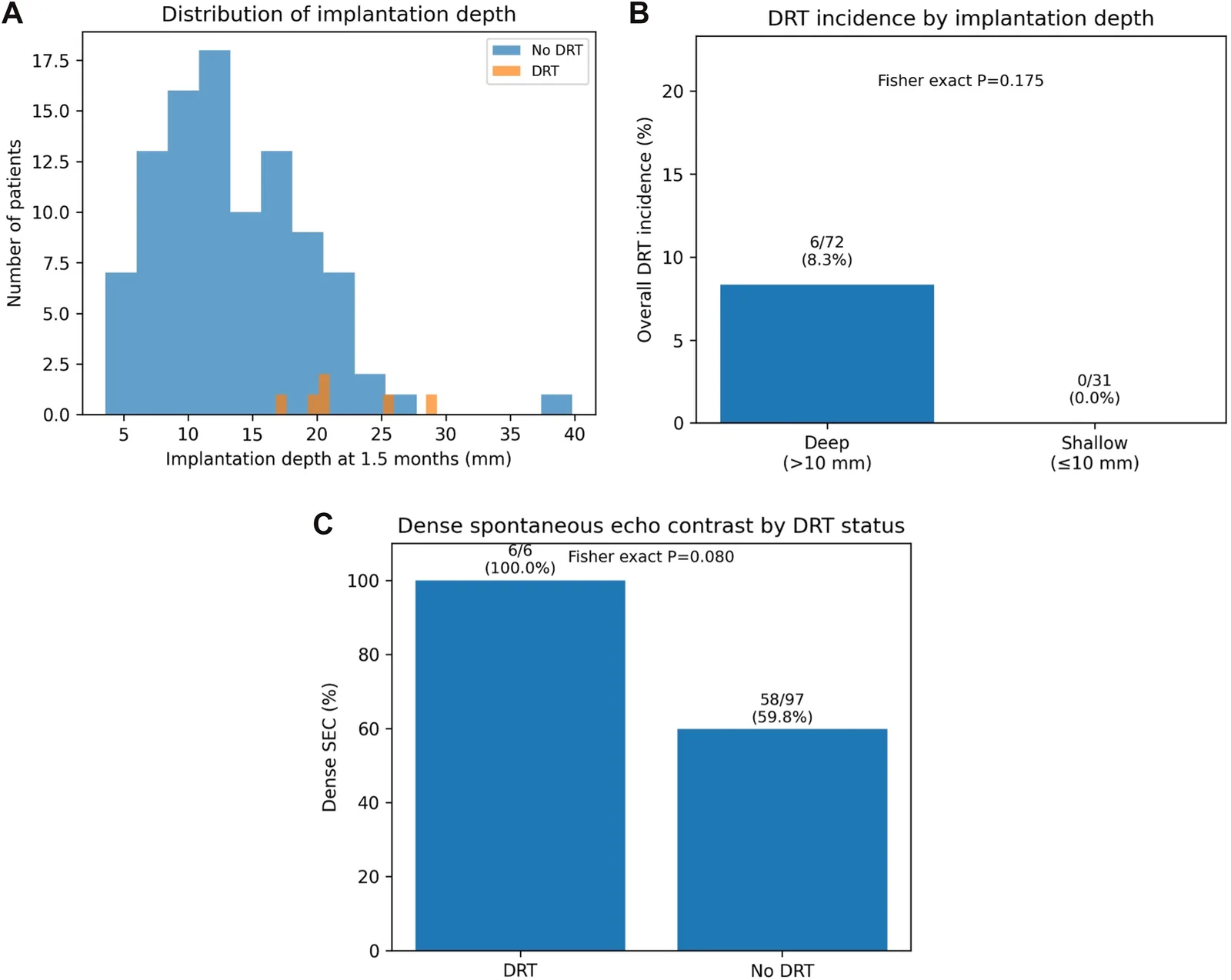
Baseline stasis phenotype and DRT. Panels display implantation depth distribution, depth categories by DRT status, and dense SEC prevalence by DRT status (see provided figure files). DRT = device-related thrombus; SEC = spontaneous echo contrast.

In an exploratory analysis restricted to patients meeting a high-stasis phenotype at baseline (deep implantation >10 mm, LAAEV ≤20 cm/s, and dense SEC; n = 29 within the landmark cohort), late/very-late DRT occurred in 4 of 12 patients (33.3%) after all-off withdrawal and in 0 of 17 (0.0%) among those maintained on any antithrombotic therapy (*P* = .021). Depth distribution and dense SEC prevalence by DRT status are presented in Figure 3.

Conversely, when restricting the comparison to patients who were all-off at the 45-day landmark (n = 33), late/very-late DRT occurred in 4 of 12 (33.3%) among those with the high-stasis phenotype vs 0 of 21 (0.0%) among those without the phenotype (*P* = .012). Given the limited number of events, receiver operating characteristic–based thresholding was not pursued and depth cut points should be interpreted cautiously.

### Safety override (continued anticoagulation despite complete sealing)

Among patients with complete sealing (PDL 0) and no DRT at the 45-day visit, 8 patients continued a direct oral anticoagulant regimen (“safety override”). The primary reason for nonwithdrawal was recent ischemic stroke within 6 months (n = 6 of 8; 75%); the remaining 2 patients had no history of ischemic stroke.

### Hemodialysis subgroup

No DRT events were observed among patients receiving maintenance hemodialysis (0 of 13). In this subgroup, oral anticoagulation was discontinued after the 45-day imaging assessment in accordance with the institutional de-escalation protocol. Given the small sample size, these findings should be interpreted cautiously.

## Discussion

### Principal findings

In this single-center registry managed with an early all-off antithrombotic protocol contingent on complete sealing, we observed 3 key findings. First, overall DRT was detected in 5.8% of patients with evaluable 45-day imaging, with 2 early and 4 late/very-late events. Second, in a landmark analysis, late/very-late DRT occurred exclusively in patients who discontinued all antithrombotic therapy at 45 days, whereas no late/very-late DRT occurred in patients maintained on any antithrombotic therapy at 45 days. Third, DRT clustered in a high-stasis phenotype characterized by greater implantation depth and baseline markers of appendage stasis (low LAAEV and dense SEC), with all DRT cases occurring in nonparoxysmal AF.

We also observed that residual PDL (any PDL >0) was common at the 45-day visit (∼55%). This likely reflects CT-based detection of very small leaks and our strict binary definition of complete sealing (PDL 0), which classifies even minimal jets as PDL positive; the clinical significance and natural history of such micro-PDL remain uncertain.

### Interpretation: The “paradox of complete sealing” as a hypothesis

A central observation of this study is that late/very-late DRT emerged after complete withdrawal of antithrombotic therapy despite documentation of complete sealing (PDL 0). We do not interpret PDL of 0 as intrinsically harmful; rather, PDL of 0 served as a key criterion for treatment de-escalation in our protocol. Our findings support a hypothesis that, when implantation depth is substantial, complete sealing may eliminate residual washout through the ostium while leaving a proximal recess (neo-appendage or cul-de-sac) exposed to severe stasis. Under pharmacologic protection, this recess may remain clinically silent; after complete withdrawal, thrombus may become detectable at later surveillance imaging. This conceptual framework—the “paradox of complete sealing”—should be considered hypothesis generating.

### Depth-dependent stasis burden and the limits of binary cutoffs

Deep implantation has been associated with DRT in previous multicenter analyses, plausibly owing to greater residual recess volume proximal to the device.^3^^,^^8^ In our cohort, the conventional binary definition of deep implantation (>10 mm) had limited discriminative value because it was common even among patients without DRT. Instead, DRT cases occurred at greater implantation depths (median ∼21 mm) and were enriched at depths of >20 mm, suggesting that DRT risk may increase with the severity of depth rather than crossing a single universal threshold. Importantly, we also observed late/very-late DRT at depths of <20 mm, arguing against a strict dichotomous cutoff and supporting a continuous, anatomy–physiology interaction model.

### Physiological substrate: Nonparoxysmal AF, SEC, and low LAAEV

DRT formation likely requires a combination of anatomic and physiological stasis. All DRT cases occurred in patients with nonparoxysmal AF and dense SEC, and baseline LAAEV was significantly lower among patients who developed DRT. These findings align with the concept that severe appendage stasis—reflecting impaired atrial mechanical function—may lower the threshold for thrombosis within a deep residual recess. Previous multicenter studies have also identified nonparoxysmal AF as a predictor of DRT after LAAC.^8^^,^^9^ In the EWOLUTION registry, dense SEC was reported as an independent predictor of DRT.^9^ In the Japanese OCEAN-LAAC registry, increasing SEC grade was independently associated with DRT, and combined stratification by SEC grade and AF type improved DRT prediction.^10^

### Therapy withdrawal, confounding by indication, and safety override

Our landmark findings should be interpreted in the context of an observational design. Patients maintained on antithrombotic therapy at 45 days may have differed systematically from those undergoing all-off discontinuation, including by physician-perceived thromboembolic risk. Notably, continuation of anticoagulation despite complete sealing was most often driven by a recent ischemic stroke within 6 months.^6^^,^^7^ Although this reflects clinically defensible caution, it introduces confounding by indication and limits causal inference regarding therapy withdrawal. Nevertheless, the absence of late/very-late DRT in the continued-therapy group, together with consistent findings in the sealing-restricted sensitivity analysis and the high-stasis phenotype subgroup, supports the possibility that complete withdrawal may unmask late/very-late DRT in selected high-stasis anatomies.

### Clinical implications

These data should not be interpreted as an argument against achieving complete sealing. Instead, they suggest that “PDL = 0” alone may be insufficient to determine suitability for early all-off withdrawal in every patient.^2^ A practical implication is the potential role of risk stratification incorporating implantation depth and baseline stasis markers. In patients with substantial implantation depth and severe stasis (dense SEC and low LAAEV), clinicians may consider (1) prolonged antithrombotic therapy, (2) stepwise de-escalation rather than abrupt cessation, and/or (3) intensified imaging surveillance beyond 45 days. Prospective validation is required before any practice change. Device size and LAA morphology were not systematically adjudicated in this registry.

### Limitations

This study has limitations inherent to single-center observational research. The number of DRT events was small, precluding multivariable adjustment and limiting precision in subgroup analyses. Follow-up imaging was CT dominant but not uniform; cardiac CT and TEE may differ in the detection of very small PDLs (micro-PDLs) and subtle thrombus, which could affect the PDL of 0 classification. PDL and DRT assessments were based on routine clinical interpretations rather than central core-laboratory adjudication. Although follow-up imaging at 6 and/or 12 months was broadly similar between landmark groups, incomplete imaging follow-up could lead to underdetection of asymptomatic late/very-late DRT. The all-off strategy is center specific and may not generalize to institutions using different post-LAAC antithrombotic regimens. Finally, the high-stasis phenotype and depth thresholds reported here should be regarded as exploratory and hypothesis generating.

## Conclusion

In a single-center cohort managed with an early all-off strategy contingent on complete sealing, late/very-late DRT occurred only after complete antithrombotic discontinuation and clustered in patients with greater implantation depth and severe baseline stasis markers. Complete sealing should remain a procedural goal; the concern highlighted by our data relates to using PDL of 0 as the sole criterion for early full antithrombotic withdrawal in patients with a high-stasis substrate. These findings motivate physiology-guided de-escalation strategies and support the need for prospective, multicenter validation.

## Declaration of generative AI and AI-assisted technologies in the writing process

During the preparation of this work, the authors used generative AI tools (ChatGPT, OpenAI; Gemini, Google) to improve language, readability, and internal consistency of the manuscript. After using these tools, the authors reviewed and edited the content as needed and take full responsibility for the content of the published article.

## Disclosures

Osamu Hashimoto has received lecture honoraria from Daiichi Sankyo, Boehringer Ingelheim, and Boston Scientific. Yasuo Tokoro has received lecture honoraria from Daiichi Sankyo and Boehringer Ingelheim. Hayata Uesako has received lecture honoraria from Boston Scientific. Yukiyoshi Tashima declares no competing interests.

## Figures and Tables

**Supplementary Figure:**
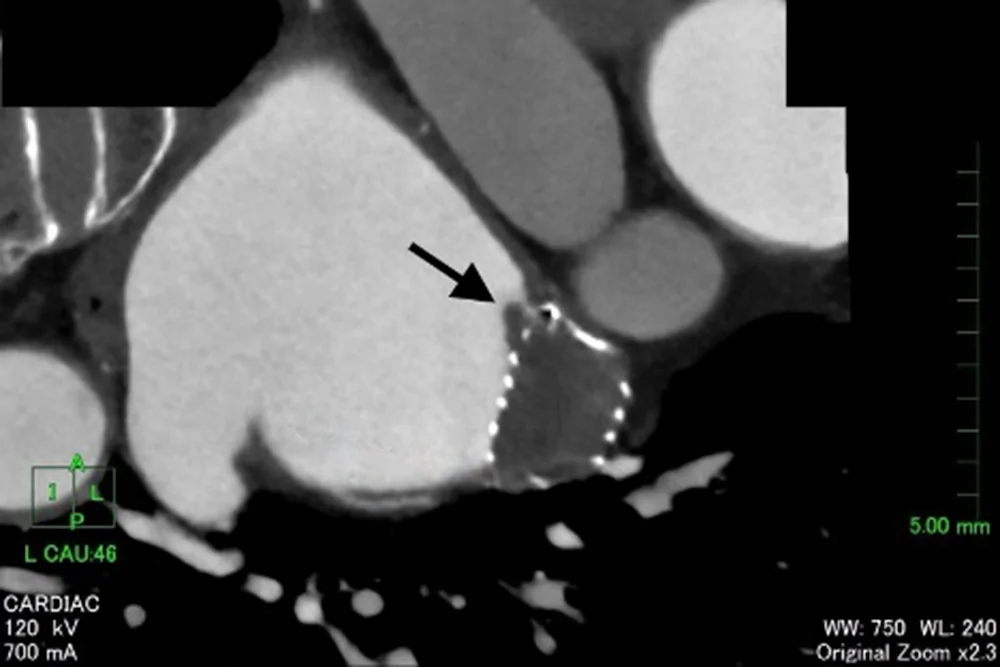


## Footnotes

Appendix Supplementary data associated with this article can be found in the online version at https://doi.org/10.1016/j.hroo.2026.04.012.
