# Supplementary material for: Late device-related thrombus after left atrial appendage closure following early antithrombotic discontinuation: The paradox of complete sealing
Source: Heart Rhythm O2. 2026 Apr 20;7(8):1462–9. doi: 10.1016/j.hroo.2026.04.012 (PMC13523577; doi:10.1016/j.hroo.2026.04.012)
Supplement: Supplementary file 1 [file mmc1.docx]

# Supplementary Material

**Figure S1. Representative CT depiction of device-related thrombus after LAAC.**

A low-attenuation mass adherent to the atrial surface of the LAAC device, consistent with device-related thrombus (DRT) as defined in the Methods, is shown (arrow). Because DRT can be eccentric, it may not be fully visualized in the standardized oblique plane used for implantation depth measurement (Figure 1); therefore, a dedicated multiplanar reconstruction plane was selected to depict the thrombus. All identifying patient information has been removed.
